# Performance of Sieve versus SwissPre Prehospital Triage Algorithms in a Simulated Mass-Casualty Incident: A Randomized Open-Label Study

**DOI:** 10.1017/S1049023X25101568

**Published:** 2025-12

**Authors:** Loric Stuby, Christophe A. Fehlmann, Salita Bellotti, Dominique Jaccard, Xavier Good, Laurent Bourgeois, Simon Regard, Laurent Suppan

**Affiliations:** 1. Genève TEAM Ambulances, Emergency Medical Services, Geneva, Switzerland; 2. Division of Emergency Medicine, Department of Acute Care Medicine, Geneva University Hospitals, Geneva, Switzerland; 3. Department of Anesthesiology, Pharmacology, Intensive Care and Emergency Medicine, Faculty of Medicine, University of Geneva, Geneva, Switzerland; 4. ESAMB-École Supérieure de Soins Ambulanciers, College of Higher Education in Ambulance Care, Conches, Switzerland; 5. A.C.E. Ambulances, Emergency Medical Services, Chêne-Bougeries, Switzerland; 6. Media Engineering Institute, School of Management and Engineering Vaud, University of Applied Sciences and Arts of Western Switzerland, Yverdon, Switzerland; 7. Cantonal Physician Division, Cantonal Health Office, State of Geneva, Geneva, Switzerland

**Keywords:** disasters, mass-casualty incidents, prehospital triage, simulation training, triage algorithms

## Abstract

**Introduction:**

Triage is an essential process used to adequately allocate resources and thus increase chances of survival in case of mass-casualty incidents (MCIs). Several triage scales are currently used, but data regarding their performance remain scarce. The objective was to compare the performance of two prehospital triage algorithms (Sieve versus SwissPre) using a validated physiological simulator.

**Methods:**

This was a web-based, randomized open-label study. A real-time evolutive simulator based on a heart-lung-brain interaction model embedding functional blocks was used to simulate the evolution of vital parameters. Participants, who were randomly allocated to either algorithm, were asked to triage 30 patients in random order. The primary outcome was the triage score (each correct decision was awarded one point). The “Immediate patients” were defined as those who would die within the first hour according to the physiological model. The secondary outcome was the duration of patient triage.

**Results:**

Out of 71 participants, 67 (94.4%) were included in the final analysis. The Sieve group achieved a mean score of 17.1 out of 30 (95%CI, 16.3 to 17.8). The SwissPre group scored 15.5 out of 30 (95%CI, 14.5 to 16.5). The mean difference between groups was 1.6 points (95%CI, 0.4 to 2.8; P = .011) in favor of the Sieve algorithm. Triage duration did not differ significantly between the Sieve (mean 43 minutes, SD = 10) and SwissPre (mean 46 minutes, SD = 23) groups, with a mean difference of three minutes (95%CI, −12 to 6; P = .507).

**Conclusions:**

The simpler Sieve algorithm may slightly outperform the more complex SwissPre in accurately categorizing critically injured patients who would likely die within 60 minutes if left untreated. No significant difference was observed in triage speed. However, these exploratory findings should be interpreted cautiously, considering the mean difference was modest and the controlled simulated setting, limiting generalizability.

## Introduction

A mass-casualty incident (MCI) is characterized by the generation of more patients than locally available resources can manage at one time. Such an event requires conceptual rearrangements and the application of specific protocols. Triage is required to better allocate the resources and increase the overall chances of survival, especially when such events impact the public health system’s essential infrastructure.^1^

Traditionally, triage allows to sieve and sort patients within minutes. It can be primary, using basic tools relying on physiological parameters such as Sort, Assess, Life-saving interventions, Treatment/triage (SALT)^2^ or modified Simple Triage and Rapid Treatment (mSTART),^3^ or it can be secondary, using more complex tools like Secondary Assessment of Victim Endpoint (SAVE).^4,5^ Unlike primary triage, SAVE incorporates additional considerations such as resource availability, expected survivability, and the likelihood of benefit from medical intervention, allowing responders to prioritize patients under conditions of scarce resources. Recently, the Spanish Prehospital Advanced Triage Method (META) has been developed.^6^ It integrates anatomical injury assessment, injury mechanism, and Advanced Trauma Life Support protocols and is organized in four phases. Due to its complexity, it is intended for use only by prehospital teams with advanced trauma training.

While primary triage is commonly viewed as one-time action, secondary triage is an on-going process, allowing to re-assess categories and take resource availability into account. There is an extensive literature on triage comparing the multiple available scales, traditionally based on under- and over-triage findings compared to the prediction of injury severity or death in specific incidents, or in simulation-based studies.^7–9^ However, there is no definitely superior scale that can be considered as a gold standard for MCI triage.^10–15^

By measuring over-triage or under-triage outcomes, most scientific articles focus on the accuracy of the different scales while often overlooking real-world applicability.^16^ Recent literature and field experiences suggests that a perfect, universal triage system is not possible since contexts and pathologies, and affected populations, vary widely. In addition, triage must also take into consideration security, environmental, logistical, and cultural issues, particularly during intentional MCIs, while keeping the humanitarian principle of impartiality at the core of the process.^17–20^ Since the acknowledgement of this unavoidable limitation is rather recent, feasibility studies are still scarce.^21^

In Switzerland, a new prehospital triage system, SwissPre, was implemented in most regions in 2023. This system was developed by a collective of several stakeholders and experts of the domain, on the basis of existing triage scales (SALT and mSTART).^22^ It was designed with the assumption that most MCIs in Switzerland would involve fires, and it therefore explicitly includes assessment of inhalation injuries. However, its complexity may require more advanced training, and to the best of the authors’ knowledge, this new scale has not yet been compared to existing and well-known triage scales such as the National Ambulance Resilience Unit Sieve Triage scale (Sieve). The aim of this study was to compare the performance of the Sieve versus the SwissPre prehospital triage algorithms in terms of triage accuracy and time efficiency using a validated physiological simulator.

## Methods

### Study Design

This was a web-based, randomized open-label study evaluating the performance of two MCI prehospital triage systems through a simulated model. The study adheres to the CONsolidated Standards Of Reporting Trials (CONSORT) statement.^23^ The CONSORT checklist is available as Supplemental Material (Table S1; available online only). The study was reviewed by the local Ethics Committee and was granted an exemption from full ethical approval (CCER, Req-2025-01174).

### Study Setting and Participants

The study was conducted on September 1, 2023, during the Prehospital Research Day in French-speaking Switzerland, a congress focusing on several themes linked to, and aiming to promote, prehospital research.^24^ All attending participants were eligible to participate. Most were paramedics, but physicians, emergency medical technicians, student paramedics, and emergency medical dispatchers were also present.

### Study Groups

Participants were randomly allocated to one of two study groups, each using a different triage algorithm.

The Sieve algorithm is a simple and rapid primary tool based on basic physiological parameters (eg, ability to walk, respiratory rate, perfusion, and consciousness) to classify patients into four priority categories (Immediate, Delayed, Minor, or Dead). Its main advantages are speed and ease of use, requiring minimal training.

The SwissPre algorithm, recently introduced in Switzerland, combines elements from established triage systems (mSTART and SALT) and includes additional parameters such as the assessment of inhalation injuries. Its strengths lie in a more comprehensive physiological assessment and improved consideration of context-specific hazards, particularly smoke inhalation injuries.

### Major Incident Patient Simulator

A web-based simulator was used to carry out this study. This program was developed on the Wegas platform^25,26^ (University of Applied Sciences of Western Switzerland; Yverdon, Switzerland) and based on HUMAn (which stands for Human is an Uncomplicated Model of Anatomy, a real-time evolutive patient model developed to simulate the evolution of vital parameters in case of major incident).^27^

The HUMAn simulator is based on a heart-lung-brain interaction model. Functional blocks are embedded in this model to simulate hemorrhages (venous and arterial) and nervous or skeletal lesions. This model simulates the evolution of major incident patients if they were left untreated after their initial injury, and was validated by prehospital experts.

### Study Sequence

On the morning of the congress, a brief plenary presentation was held. The aim was to inform the participants that this study was going to take place during the congress, to present the study team, and to give them the opportunity to ask questions and enroll if they wished to. Participation was voluntary, and no incentive was provided.

Participants were admitted in groups of up to ten individuals in a dedicated room equipped with computers. They were given further information regarding the study and gave their informed consent, which was then electronically recorded. It was emphasized that it was the algorithm, not their personal skills, that was being assessed. The following instructions were then provided:

1. The aim was not to perform the prehospital triage according to personal instinct or to usual algorithm, but to scrupulously follow the algorithm to which they had been randomized;
2. If the patient was standing, it was implied that they were capable to walk;
3. If the patient was sitting down, participants had to press the speech bubble and ask whether they could walk;
4. To apply a tourniquet, the tourniquet icon had to be clicked before selecting where it had to be placed on the patient’s body;
5. After each action, it was necessary to assess its effect (it was for example necessary to re-assess the respiratory rate after inserting a nasopharyngeal cannula in a person who was initially not breathing);
6. The text displayed in the upper left corner of the screen contained important information about the patient and had to be taken into account; and
7. External hemorrhages had to be quantified as either minor or major.

This information was also displayed on a board visible to everyone, which was present throughout the exercise.

Each participant then received a sealed envelope containing the triage algorithm to be used, as well as unique identifiers for logging into the system. After opening the envelop, but before starting the exercise, participants were given a maximum of five minutes to familiarize themselves with the algorithm they had drawn, which remained available throughout the exercise. They were then asked to log in to the study platform, electronically signed the consent form, and completed a demographic questionnaire before being re-directed to the simulator. All data were recorded electronically.

**Figure 1.**
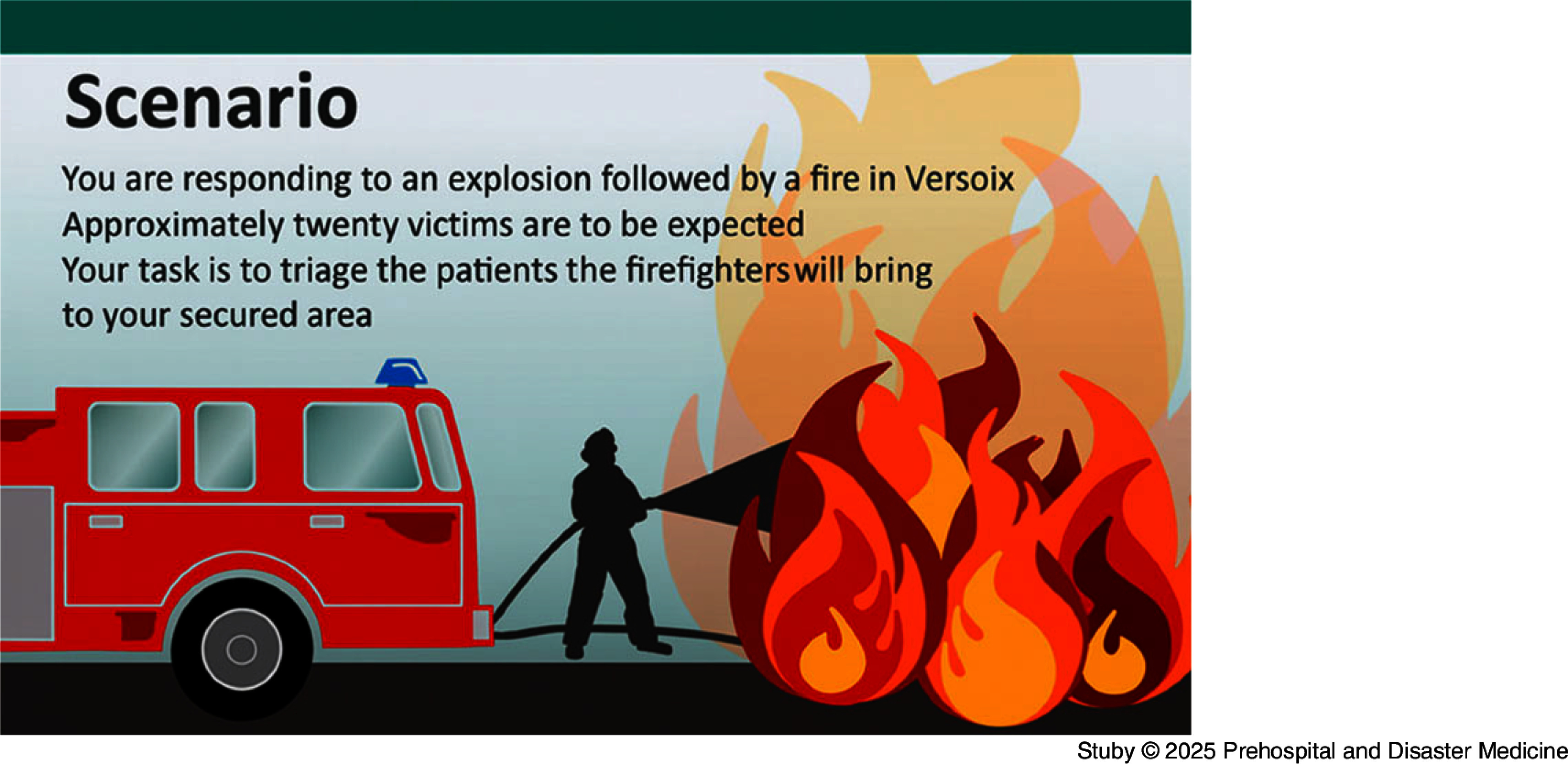
Description of the Situation.

To remain consistent with the prehospital triage situations most often encountered in Switzerland, this study was based on a fire situation with multiple victims. The situation was displayed on a single slide (Figure 1). A splash screen detailing the rules used by the simulator was then shown (Supplemental Material Figure S2; available online only). After this introduction, each participant was asked to triage 30 patients (all vignettes are provided as Supplemental Material File S3; available online only), in random order, and without any prior training on the simulator. Once all patients were triaged, participation was ended with no feedback or other teaching intervention. Participants were aware that the objective was to assess a triage algorithm, but they were blinded as to the precise study outcomes. They were also asked not to share their experience with congress attendants who had not yet taken part in the study.

### Randomization and Concealment of Allocation

To ensure balance between the groups, block randomization was generated by first author, with a 1:1 allocation ratio and a block size of ten. No one else had access to the randomization list prior to the conduct of the study. As the participant discovered the algorithm to be applied by opening an opaque, sealed envelope, the study was necessarily “open.” However, to minimize bias as much as possible, allocation was revealed as late as possible (ie, after registration, initial information, and the resolution of all remaining questions). From the moment the allocation was known, no further contact was allowed with the study team or other participants. Bias was further minimized by the random order in which the simulated patients were presented.

### Outcomes and Statistical Analysis

As the primary goal was to compare the capacity of each algorithm to differentiate patients who needed immediate treatment from others, the primary outcome was the triage score. Since patients were simulated by the validated HUMAn model, their outcome was determined by the evolution of their physiological parameters according to the impact of their injuries.

Participants received one point for a correct triage decision and zero points for an incorrect decision. Triage was considered correct only if:

1. Not already dead but death ≤60 minutes and categorized “Priority 1 – Immediate” (red coded);
2. Death >60 minutes and categorized either “Priority 2 – Urgent” (yellow coded), “Priority 3 – Delayed” (green coded), or “Priority 4 – Involved but unharmed” (white coded); or
3. Already dead and categorized “Dead” (black coded).

Thus, the triage score could range from zero (all incorrect triage decisions) to 30 (all correct triage decisions). The gold standard was established based on the validated physiological model. Patients triaged in the “Immediate” category were defined as those who, if left untreated, would have died within the first hour according to the physiological evolution computed by the HUMAn model.^28^ Since the 60-minute threshold is a commonly used operational references rather than a precisely evidence-based cut-off, sensitivity analyses of the primary outcome were conducted using two additional cut-offs (90 and 120 minutes) to assess how varying the threshold would affect triage performance.

The secondary outcome measured the duration of patient triage, defined as the total time spent on the simulator, including any therapeutic interventions.

A formal sample size calculation was not performed as the study used a convenience sample. Participants’ characteristics were described using descriptive statistics, expressed as mean and standard deviation (SD) or 95% confidence interval (95%CI) for continuous variables and frequency with relative percentage for categorical variables. For the outcomes, two-sided t-tests were conducted to assess differences between the two groups. Additionally, prespecified subgroup analyses explored potential interactions based on various provider characteristics (gender, age, experience, and disaster management training), through linear regression models including interaction terms. These variables were dichotomized at their median values. The width of the 95% confidence intervals for secondary and subgroup analyses has not been adjusted for multiplicity, and they should not be used in place of hypothesis testing. P values < .05 were considered significant. All analyses were performed with Stata version 17 (StataCorp LLC; College Station, Texas USA).

## Results

### Participants

**Figure 2.**
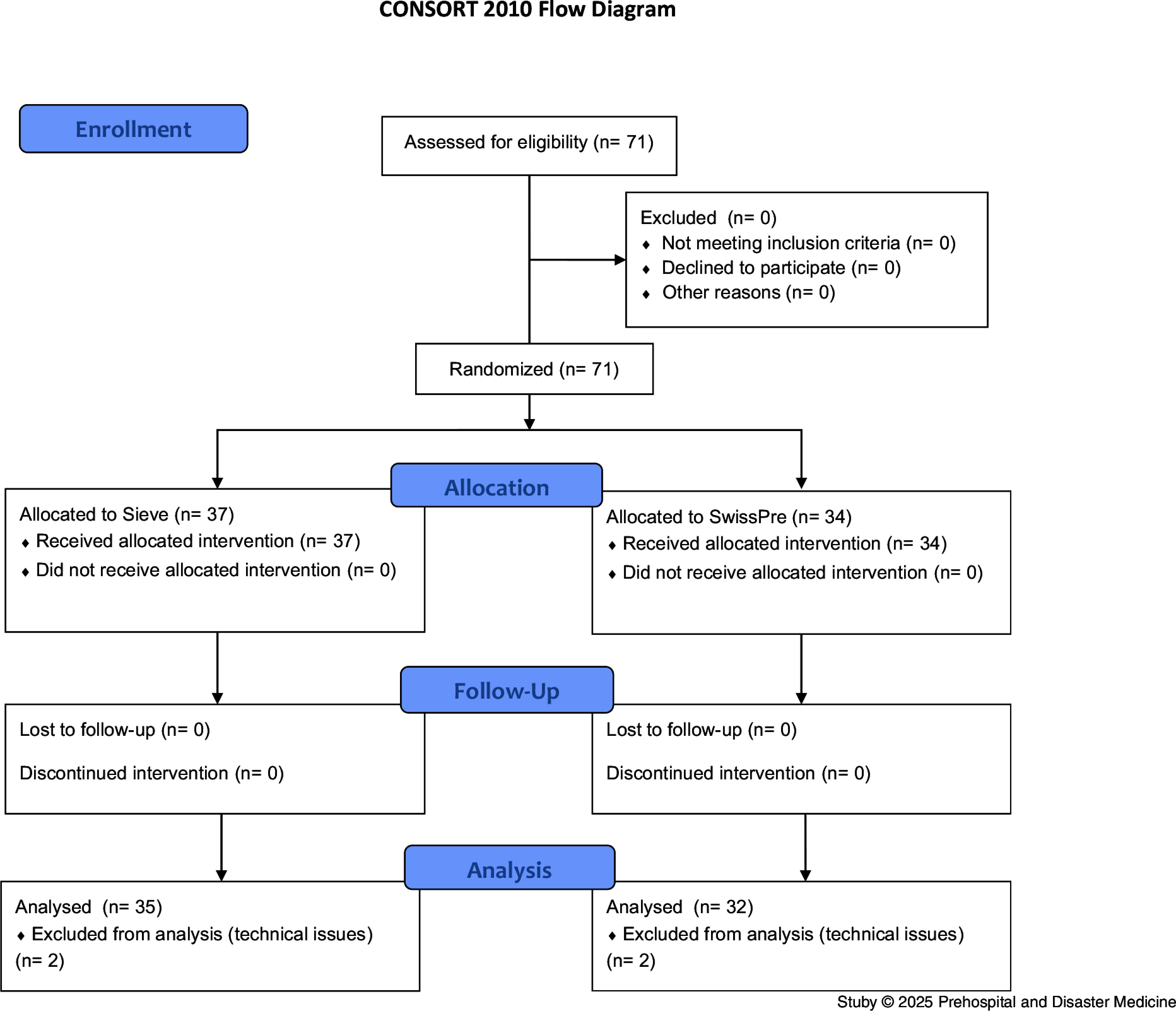
Flowchart.

A total of 71 health care providers participated in the simulation and were randomized into two groups, of which four participants (5.6%) were excluded from the analysis due to technical issues, namely inconsistent simulator behavior (Figure 2). The characteristics of the participants included in the final dataset are detailed in Table 1. Each participant performed triage for 30 victims, resulting in a total of 2,010 victims being assigned to a triage category.

**Table 1.** Participant Characteristics

	**Sieve (n = 35)**	**SwissPre (n = 32)**
Gender – n (%)		
Women	14 (40.0)	7 (21.9)
Men	21 (60.0)	25 (78.1)
Age – mean (SD)	34.8 (SD = 6.6)	35.8 (SD = 8.8)
Age Category – n (%)		
<35 Years	19 (54.3)	15 (46.9)
>34 Years	16 (45.7)	17 (53.1)
Work Experience – mean (SD)	9.9 (SD = 6.9)	9.8 (SD = 8.1)
Work Experience Category – n (%)		
<10 Years	17 (48.6)	17 (53.1)
>9 Years	18 (51.4)	15 (46.9)
Previous Training in Disaster Management – n (%)	14 (40.0)	18 (56.3)
Workplace Region – n (%)		
Geneva	16 (45.7)	15 (46.9)
Neuchâtel/Fribourg	8 (22.9)	7 (21.9)
Vaud	4 (11.4)	6 (18.8)
Ticino/Valais	7 (20.0)	4 (12.5)

### Primary Outcome

**Figure 3.**
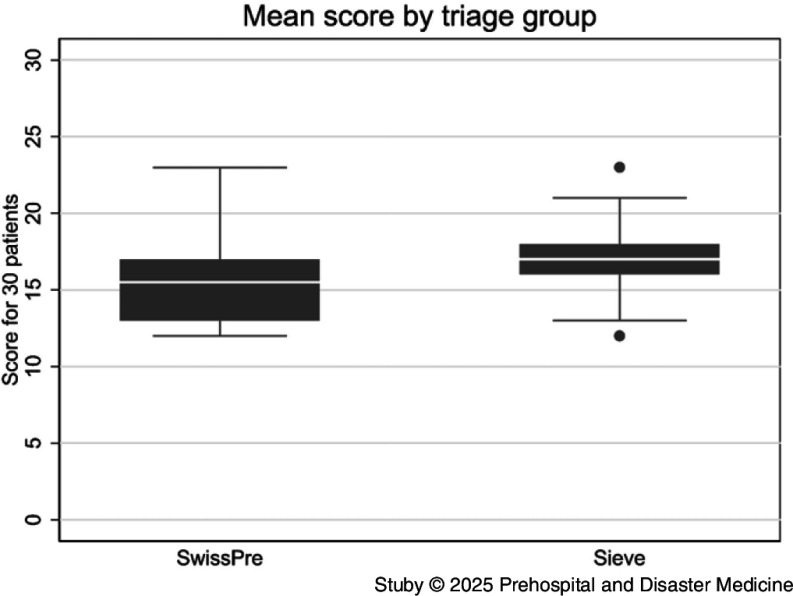
Mean Score by Triage Tool Group.

**Figure 4.**
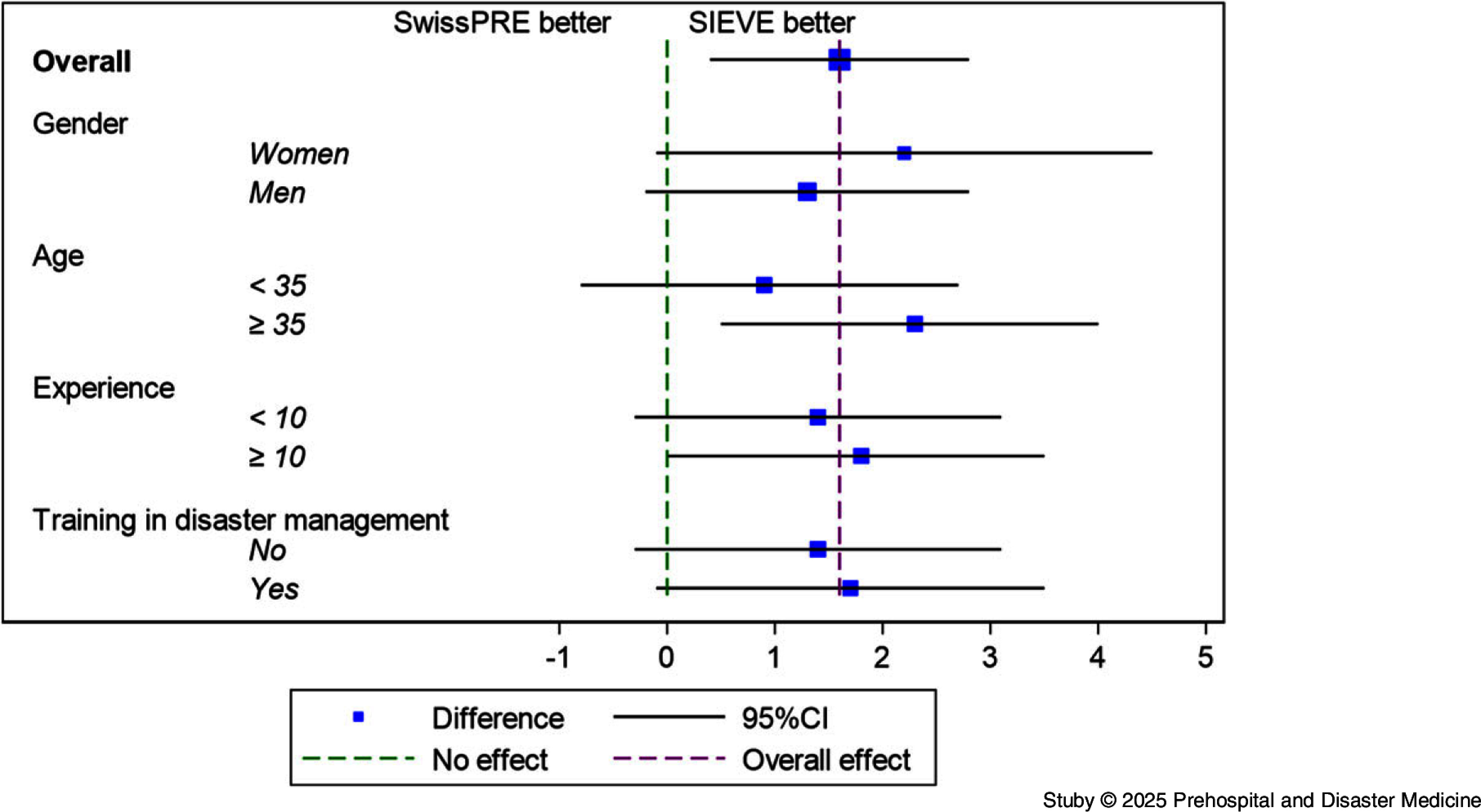
Prespecified Subgroups Analyses.

The Sieve group achieved a mean score of 17.1 out of 30 (95%CI, 16.3 to 17.8). The SwissPre group scored 15.5 out of 30 (95%CI, 14.5 to 16.5). The mean difference between groups was 1.6 points (95%CI, 0.4 to 2.8; P = .011; Figure 3) in favor of the Sieve algorithm. Subgroup analysis indicated that the modest advantage of the Sieve algorithm over SwissPre was consistent across participant characteristics, including gender, age, work experience, and prior disaster management training (Figure 4). All subgroups showed overlapping 95% CI with the overall effect, suggesting that these characteristics did not materially influence triage accuracy.

### Secondary Outcome

Triage duration did not differ significantly between the Sieve (mean 43 minutes, SD = 10) and SwissPre (mean 46 minutes, SD = 23) groups, with a mean difference of three minutes (95%CI, –12 to 6; P = .507).

### Sensitivity Analyses

The post hoc sensitivity analyses showed a slight difference with the 90-minute cutoff (difference = 1.6; 95%CI, 0.6 to 2.7) in favor of the Sieve algorithm. However, with the 120-minute cutoff, the effect disappeared (difference = 0.6; 95%CI, −3.0 to 1.4).

## Discussion

### Main Considerations

This study evaluated the performance of the Sieve and the SwissPre triage algorithms in a simulated MCI. The Sieve algorithm achieved a slightly higher triage accuracy than SwissPre, despite the latter having been explicitly developed for fire-related incidents, such as the one simulated. This suggests that Sieve may be more effective in correctly categorizing patients based on their risk of mortality within 60 minutes.

Although the mean difference in triage accuracy between the two algorithms was 1.6 points and reached statistical significance, this difference may be of limited clinical relevance. Each correct triage decision was scored as one point, so the observed difference represents a relatively modest improvement in overall performance. In practical terms, while statistically detectable, this effect size may not substantially alter patient outcomes in real-life MCIs.

No difference was observed in the duration of triage between the two groups. This study was not designed nor powered to detect differences in this secondary outcome.

Sensitivity analyses supported the robustness of the primary outcome but revealed that the advantage of Sieve disappeared when the critical mortality window was extended to 120 minutes. This finding is consistent with the fundamental aim of primary triage, which is to rapidly identify “Immediate” patients, those at the highest risk of death without prompt intervention. Accordingly, the Sieve’s superiority appears most relevant for detecting these critically injured patients, while its advantage decreases when considering less time-sensitive cases.

These findings align with previous research suggesting that no single triage system is universally superior, as accuracy and applicability often depend on the specific context and event characteristics.^29^ Prior studies have highlighted the limitations of physiologically based triage models, particularly in recognizing occult injuries and accounting for logistical and environmental constraints.^30,31^ The current study contributes to this discussion by demonstrating that while Sieve may offer slightly better immediate prioritization, its advantage diminishes as the time horizon for critical deterioration extends. This is consistent with literature emphasizing that triage performance is influenced by both patient evolution and algorithm rigidity.^32,33^

### Perspectives

Future studies should focus on validating these findings in real-world settings, where dynamic factors such as responder fatigue, evolving triage conditions, and logistical constraints come into play. Additionally, reviews from actual MCIs could offer valuable insights into the practical application, limitations, and adaptability of various triage algorithms in high-pressure environments. Given the minimal differences in performance and the absence of a clear time-saving advantage, decisions regarding the adoption of a particular algorithm should also consider factors like ease of training, usability in high-stress situations, and its ability to adapt to real-world complexities.

## Strengths and Limitations

Several limitations should be acknowledged. First, while the simulation model provided a controlled testing environment, it does not fully replicate the complexities of real-world MCIs, where factors like environmental hazards, patient movement, and rescuer stress can significantly influence triage decisions. Second, the open-label design could have introduced bias, as participants were aware of the algorithm they were using, potentially influencing their decision making. However, efforts to minimize bias were made by randomizing the patient case order and delaying algorithm allocation until the last possible moment. Additionally, four participants were excluded due to technical issues, which, although a small proportion, may have had some impact on the overall findings. Both algorithms may perform differently in different scenarios. While the simulation scenario involved a fire (which could theoretically have favored the SwissPre algorithm, as it was originally developed with the assumption the most MCIs in Switzerland would involve fires), the results instead showed an advantage for the Sieve algorithm. Nevertheless, potential scenario-related bias cannot be completely ruled out. Moreover, the study population consisted mostly of paramedics attending a conference, which might not reflect the general skill distribution of field providers. To mitigate potential performance bias, all participants received standardized instructions, the order of patient cases was randomized, and algorithm allocation was delayed until the last possible moment. These measures ensured that participants’ performance reflected algorithm characteristics rather than familiarity or expectation bias. Lastly, patients were not designed specifically to test both scales and their specificities. For example, no patient had a stridor, which would be tagged as red (Priority 1 – Immediate) in SwissPre but would not been taken into consideration with Sieve.

Despite these limitations, several strengths should be acknowledged. The study employed a randomized design within a controlled simulation environment, ensuring standardized exposure to both triage algorithms. The web-based simulator provided consistent patient presentations alongside a dynamic human simulation model. Unlike static vignettes, this model allowed for the real-time evolution of patient conditions, providing more nuanced and clinically relevant data that better reflect the complexities of real-world triage scenarios.

## Conclusion

The simpler Sieve algorithm may slightly outperform the more complex SwissPre in accurately categorizing critically injured patients who would likely die within 60 minutes if left untreated. However, this exploratory finding should be interpreted cautiously, considering the mean difference was modest and the controlled simulated setting, limiting generalizability to real-world MCIs. No significant difference was observed in triage speed. These findings contribute to the on-going discussion regarding the optimal triage approach for MCIs, emphasizing the need for further research to address the practical challenges of triage implementation in real-world settings.

## Supporting information

Stuby et al. supplementary material Stuby et al. supplementary material

## Supplementary Materials

To view supplementary material for this article, please visit https://doi.org/10.1017/S1049023X25101568
