# Supplementary figures and images for: Performance of Sieve versus SwissPre Prehospital Triage Algorithms in a Simulated Mass-Casualty Incident: A Randomized Open-Label Study
Source: Prehosp Disaster Med. 2025 Dec 11;40(6):292–8. doi: 10.1017/S1049023X25101568 (PMC12818972; doi:10.1017/S1049023X25101568)

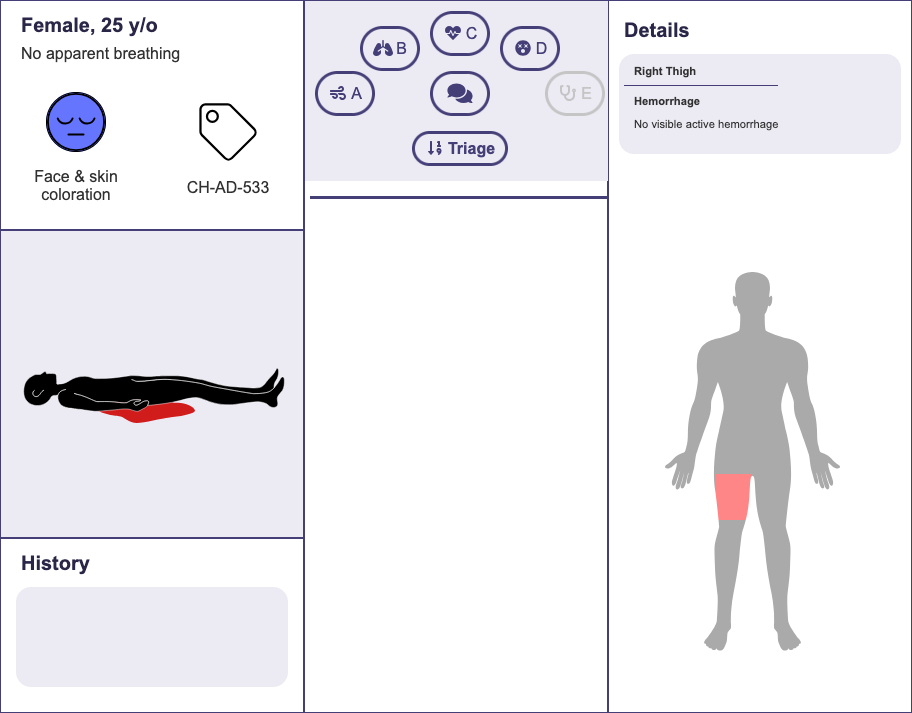

Supplement: Stuby et al. supplementary material [file S1049023X25101568sup001.zip › CH-AD-533.png]

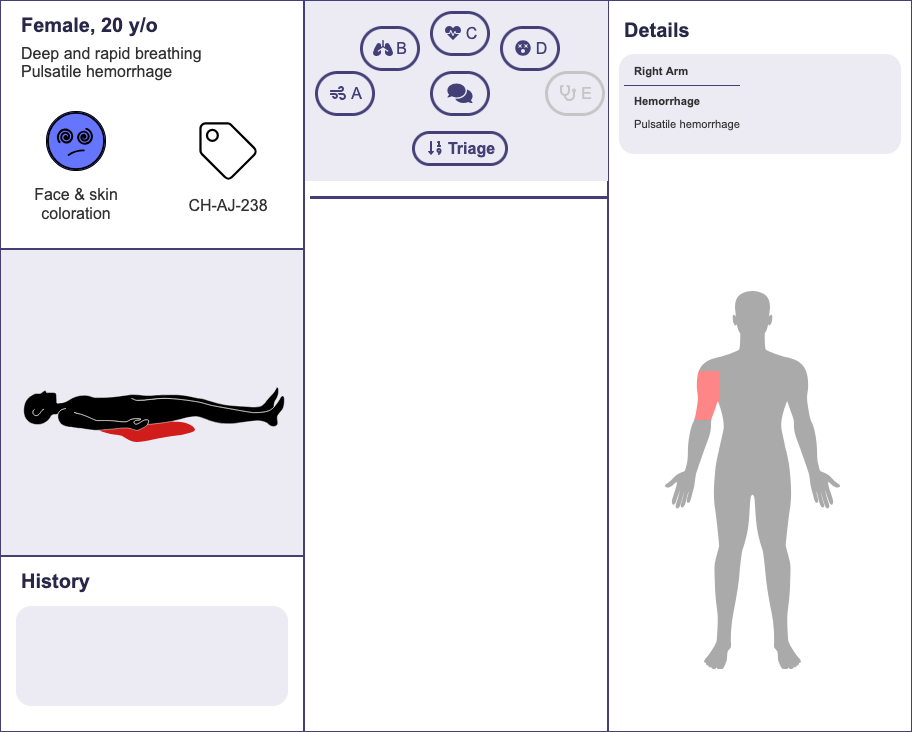

Supplement: Stuby et al. supplementary material [file S1049023X25101568sup001.zip › CH-AJ-238.png]

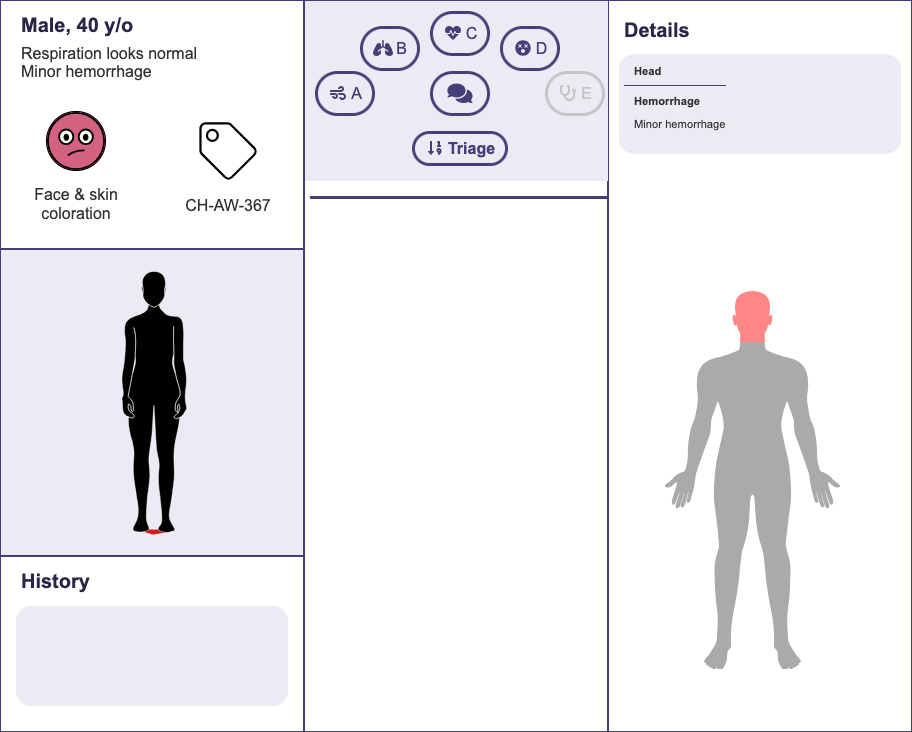

Supplement: Stuby et al. supplementary material [file S1049023X25101568sup001.zip › CH-AW-367.png]

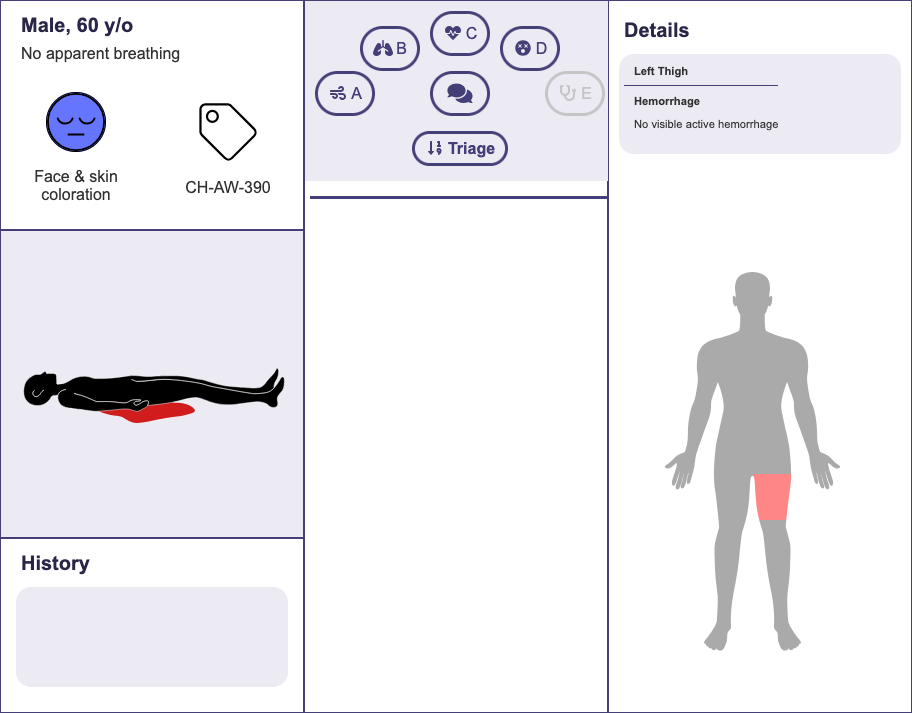

Supplement: Stuby et al. supplementary material [file S1049023X25101568sup001.zip › CH-AW-390.png]

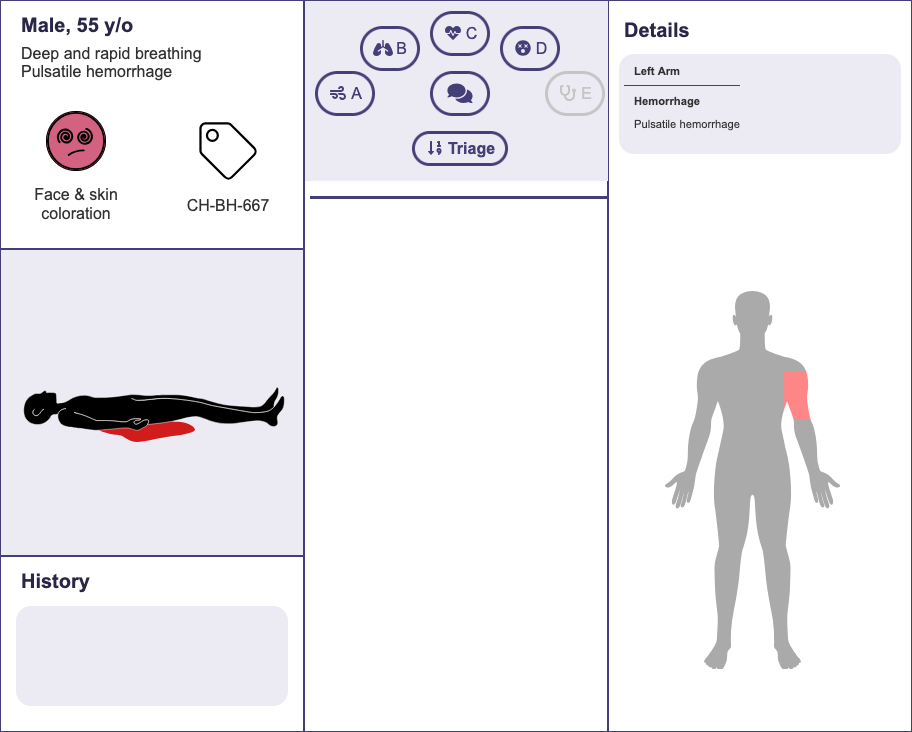

Supplement: Stuby et al. supplementary material [file S1049023X25101568sup001.zip › CH-BH-667.png]

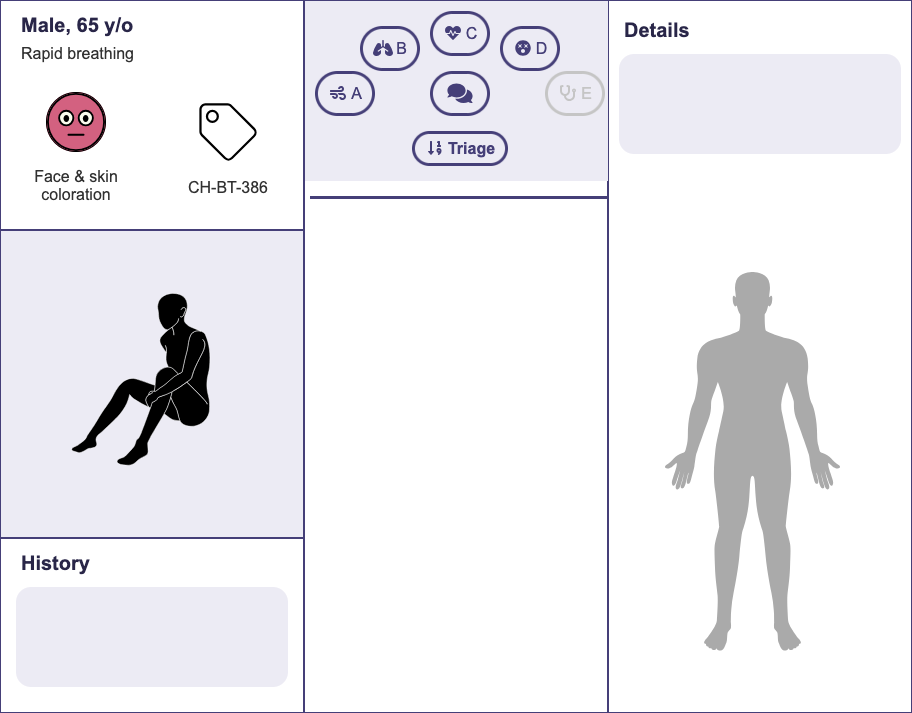

Supplement: Stuby et al. supplementary material [file S1049023X25101568sup001.zip › CH-BT-386.png]

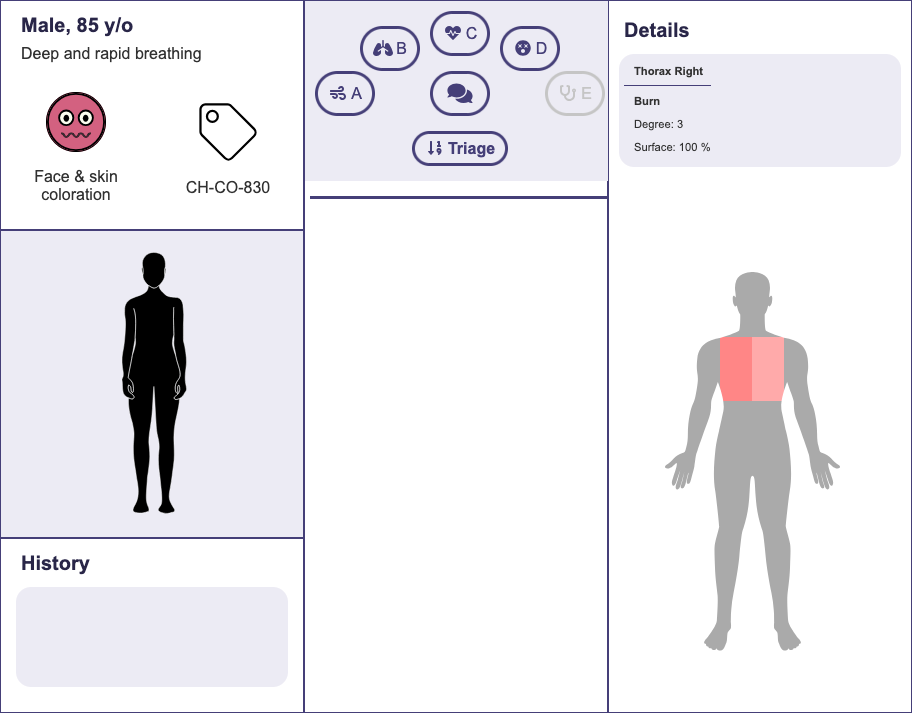

Supplement: Stuby et al. supplementary material [file S1049023X25101568sup001.zip › CH-CO-830.png]

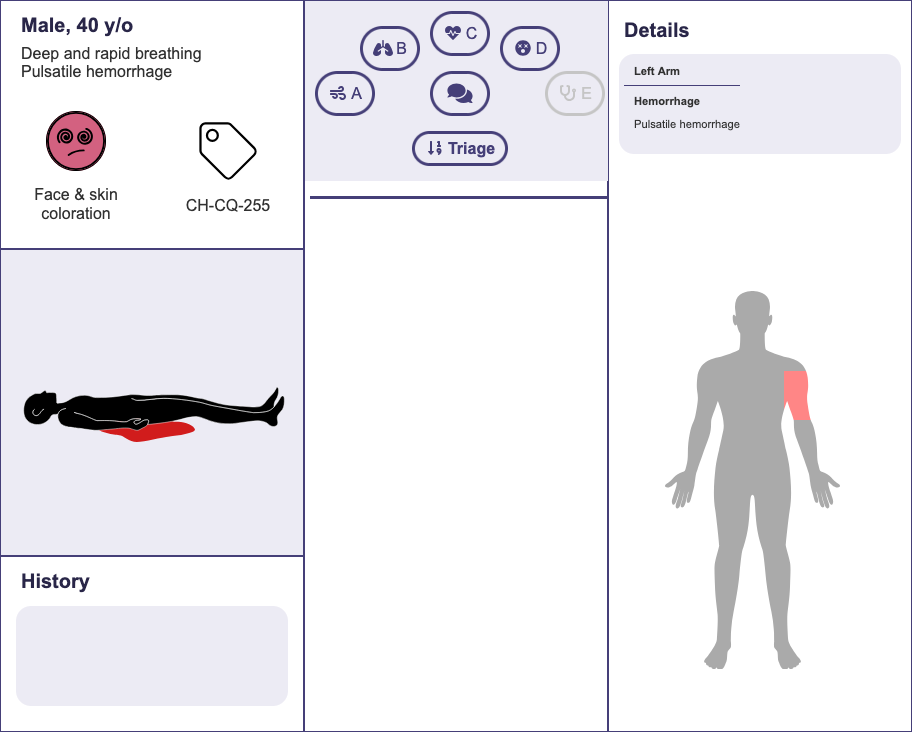

Supplement: Stuby et al. supplementary material [file S1049023X25101568sup001.zip › CH-CQ-255.png]

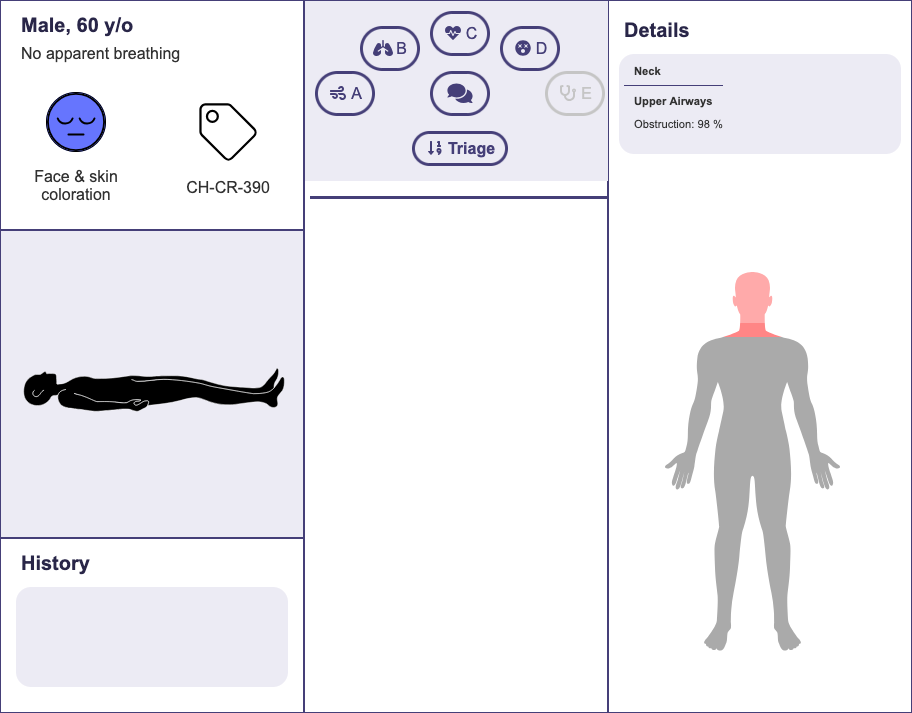

Supplement: Stuby et al. supplementary material [file S1049023X25101568sup001.zip › CH-CR-390.png]

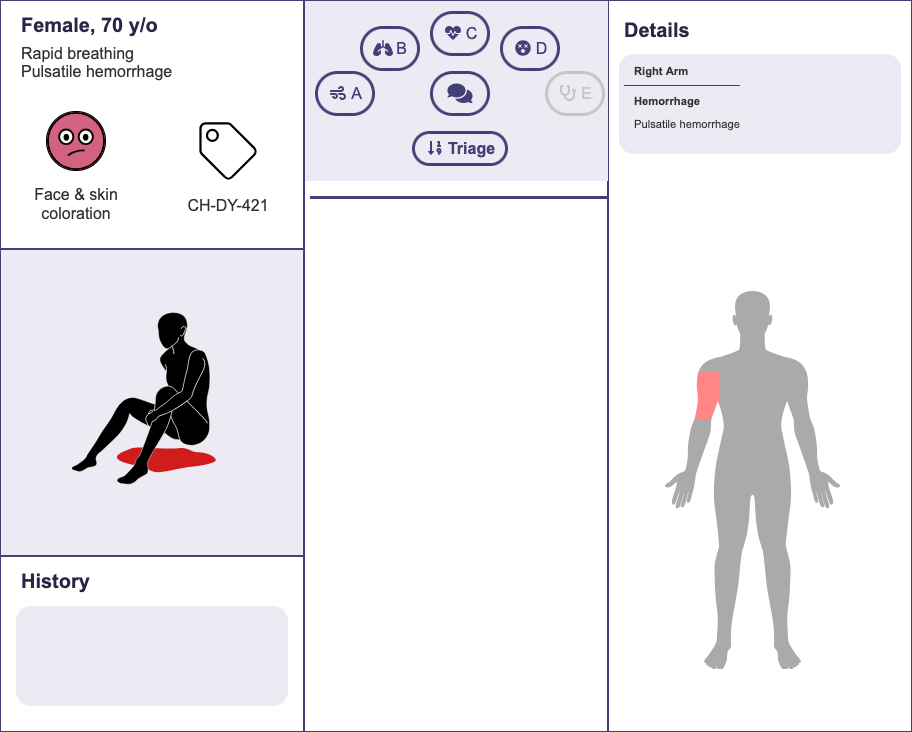

Supplement: Stuby et al. supplementary material [file S1049023X25101568sup001.zip › CH-DY-421.png]

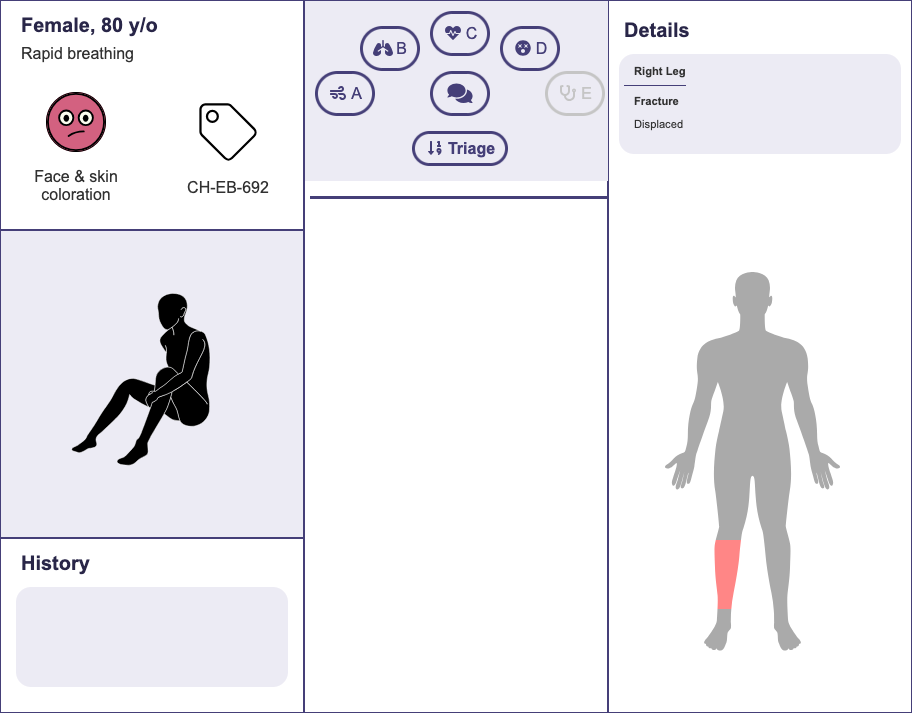

Supplement: Stuby et al. supplementary material [file S1049023X25101568sup001.zip › CH-EB-692.png]

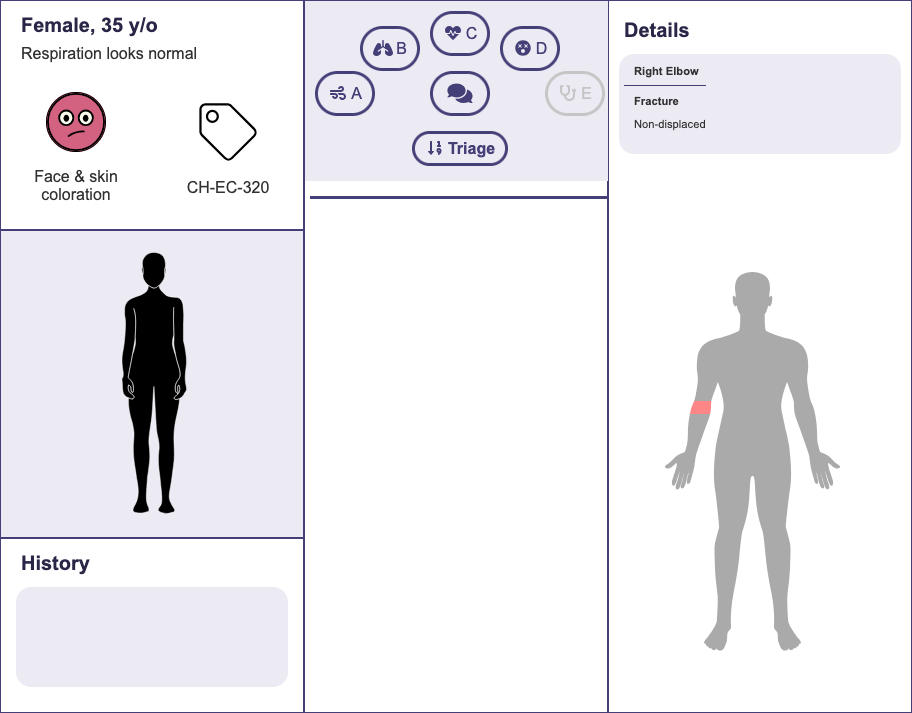

Supplement: Stuby et al. supplementary material [file S1049023X25101568sup001.zip › CH-EC-320.png]

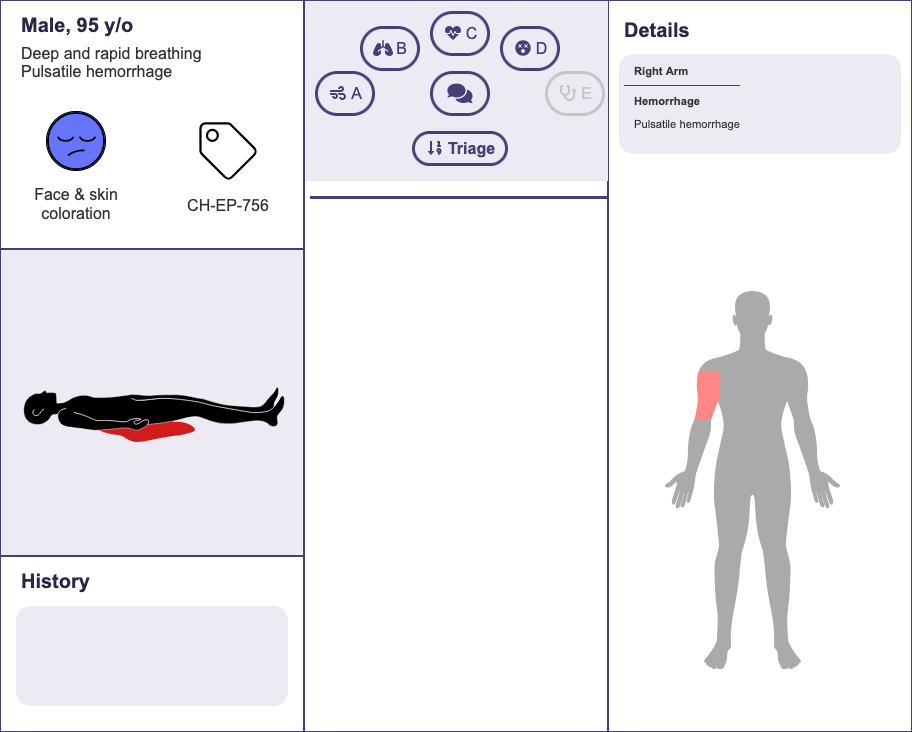

Supplement: Stuby et al. supplementary material [file S1049023X25101568sup001.zip › CH-EP-756.png]

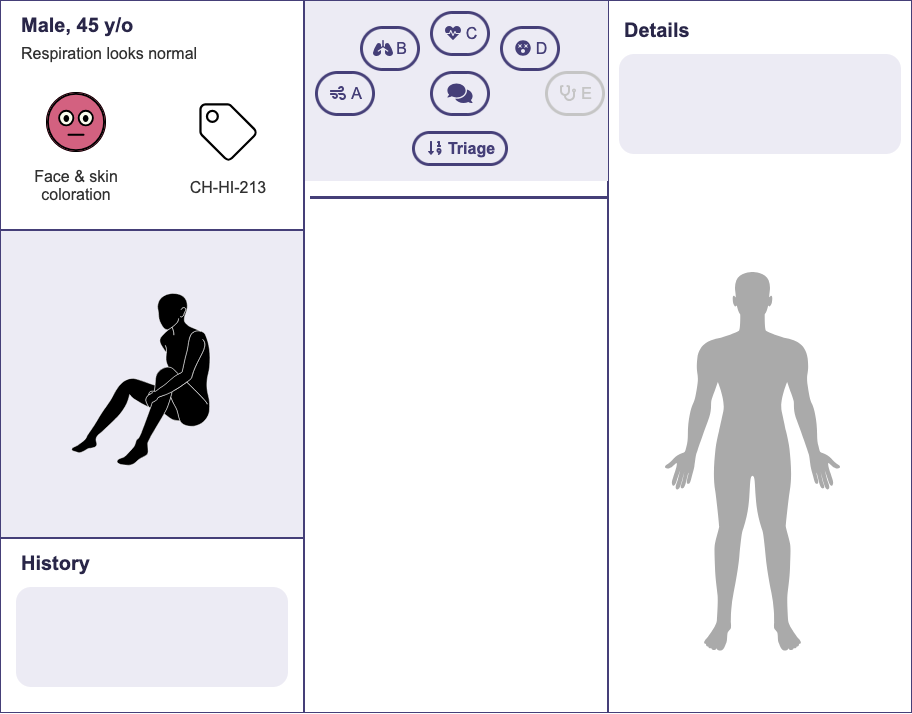

Supplement: Stuby et al. supplementary material [file S1049023X25101568sup001.zip › CH-HI-213.png]

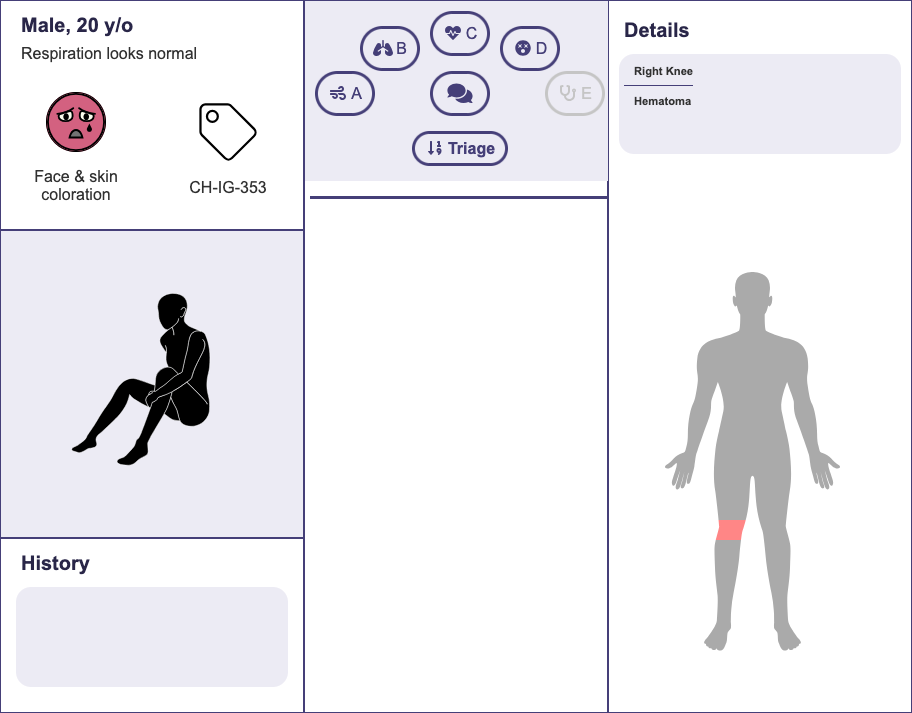

Supplement: Stuby et al. supplementary material [file S1049023X25101568sup001.zip › CH-IG-353.png]

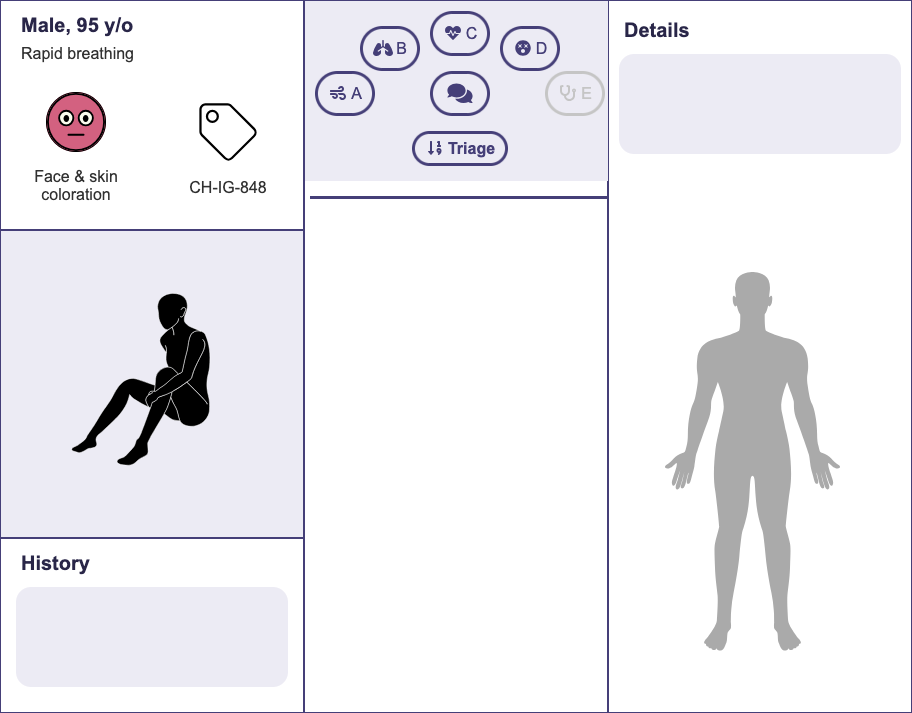

Supplement: Stuby et al. supplementary material [file S1049023X25101568sup001.zip › CH-IG-848.png]

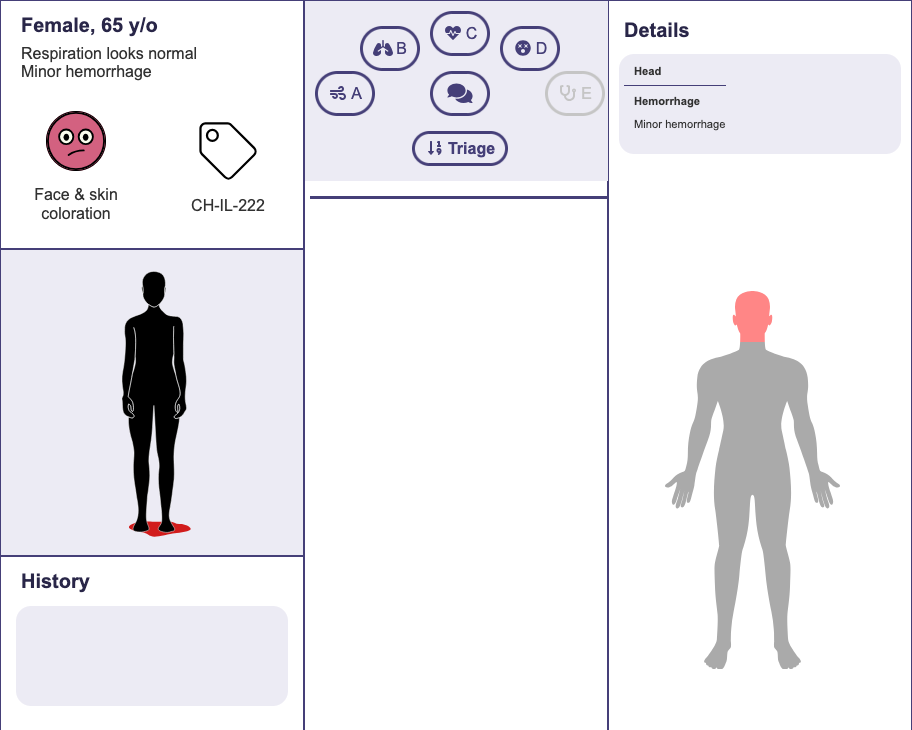

Supplement: Stuby et al. supplementary material [file S1049023X25101568sup001.zip › CH-IL-222.png]

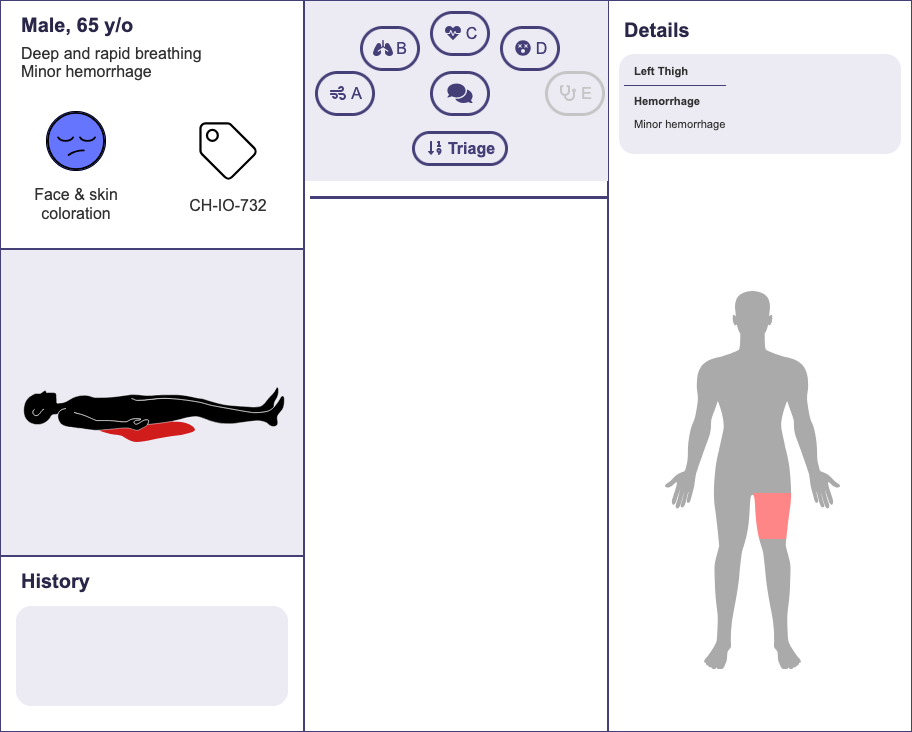

Supplement: Stuby et al. supplementary material [file S1049023X25101568sup001.zip › CH-IO-732.png]

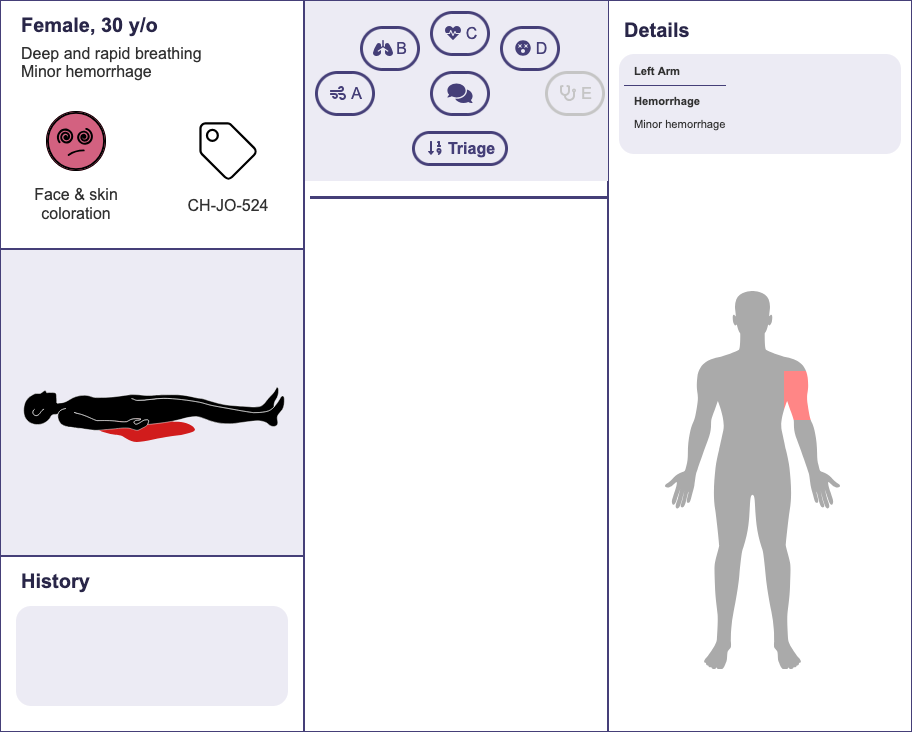

Supplement: Stuby et al. supplementary material [file S1049023X25101568sup001.zip › CH-JO-524.png]

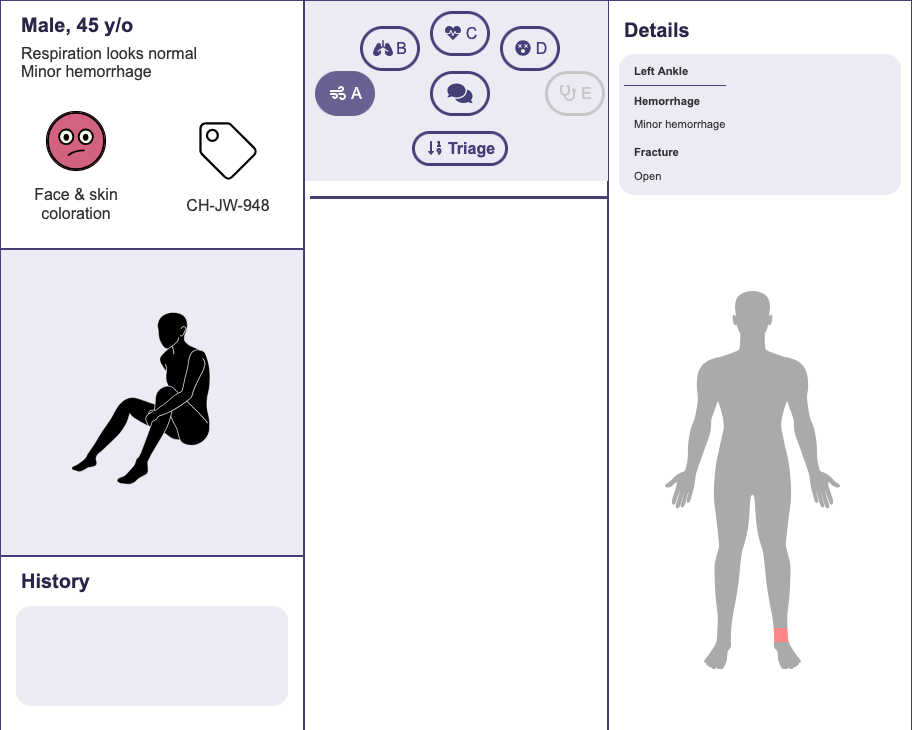

Supplement: Stuby et al. supplementary material [file S1049023X25101568sup001.zip › CH-JW-948.png]

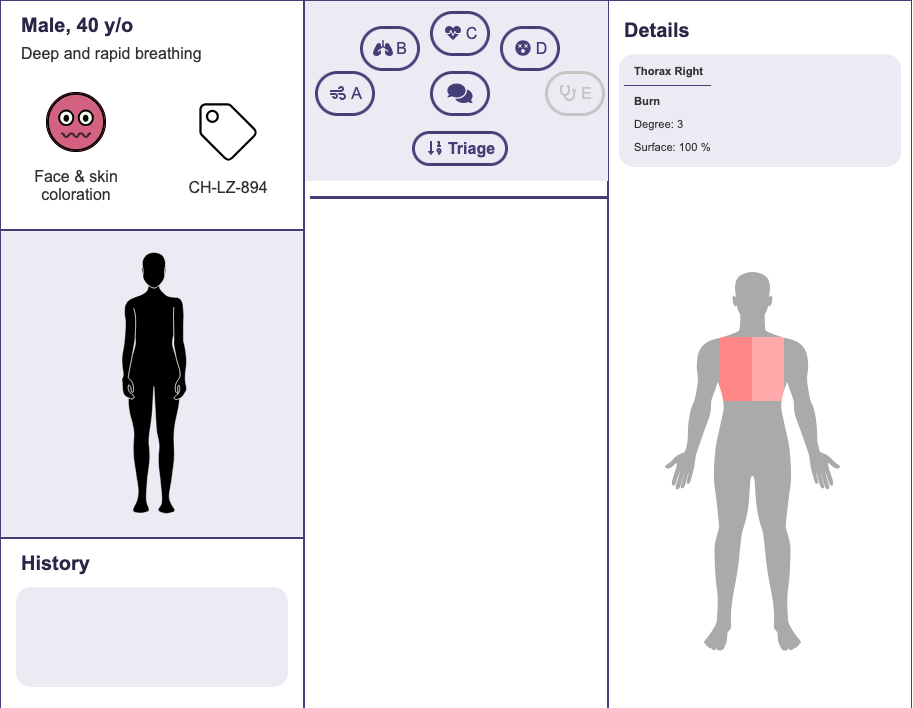

Supplement: Stuby et al. supplementary material [file S1049023X25101568sup001.zip › CH-LZ-894.png]

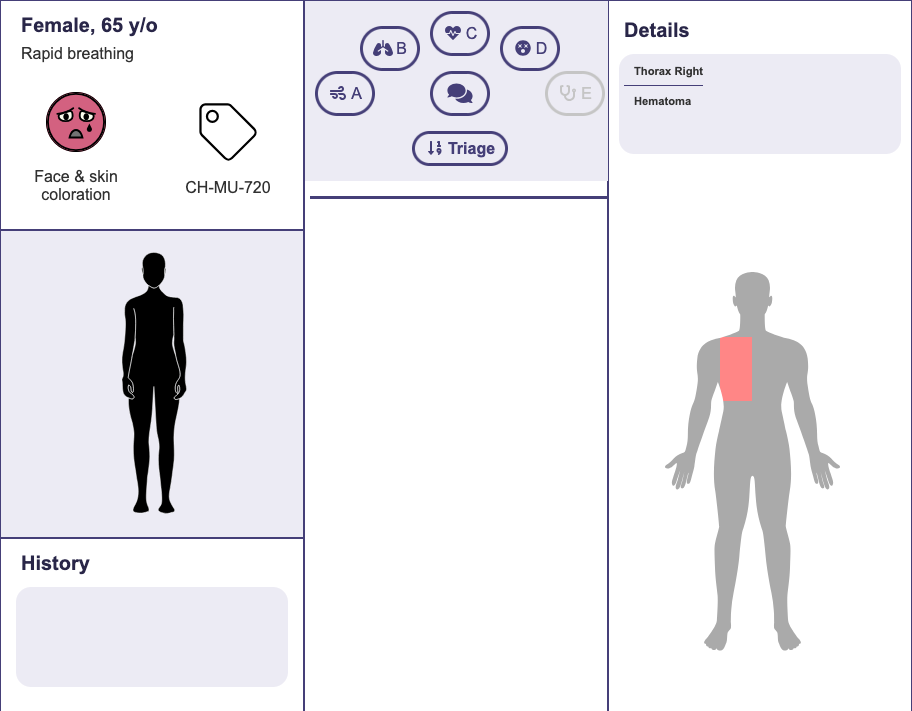

Supplement: Stuby et al. supplementary material [file S1049023X25101568sup001.zip › CH-MU-720.png]

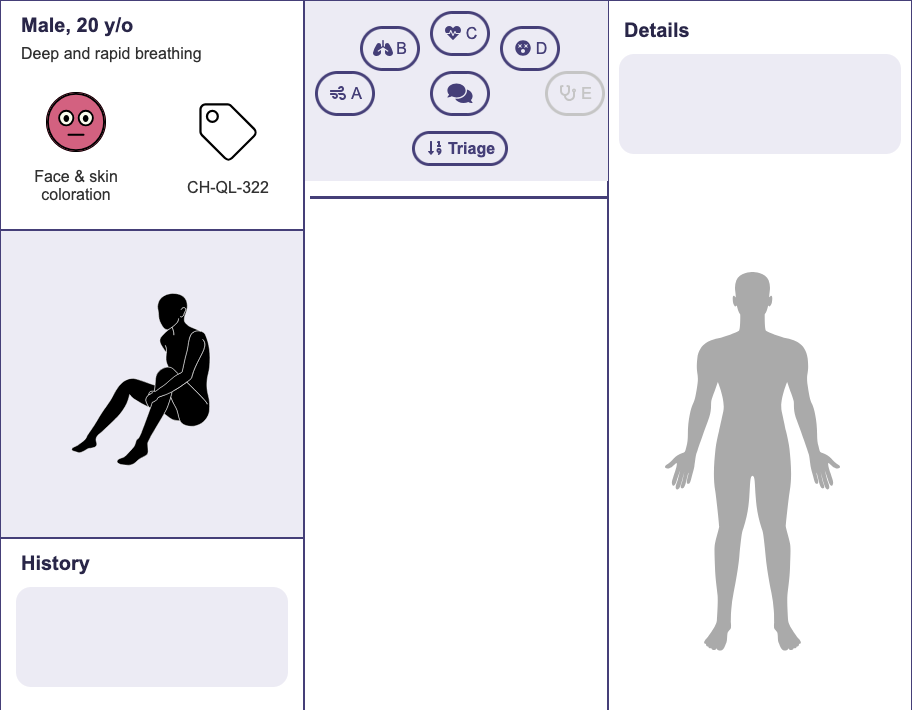

Supplement: Stuby et al. supplementary material [file S1049023X25101568sup001.zip › CH-QL-322.png]

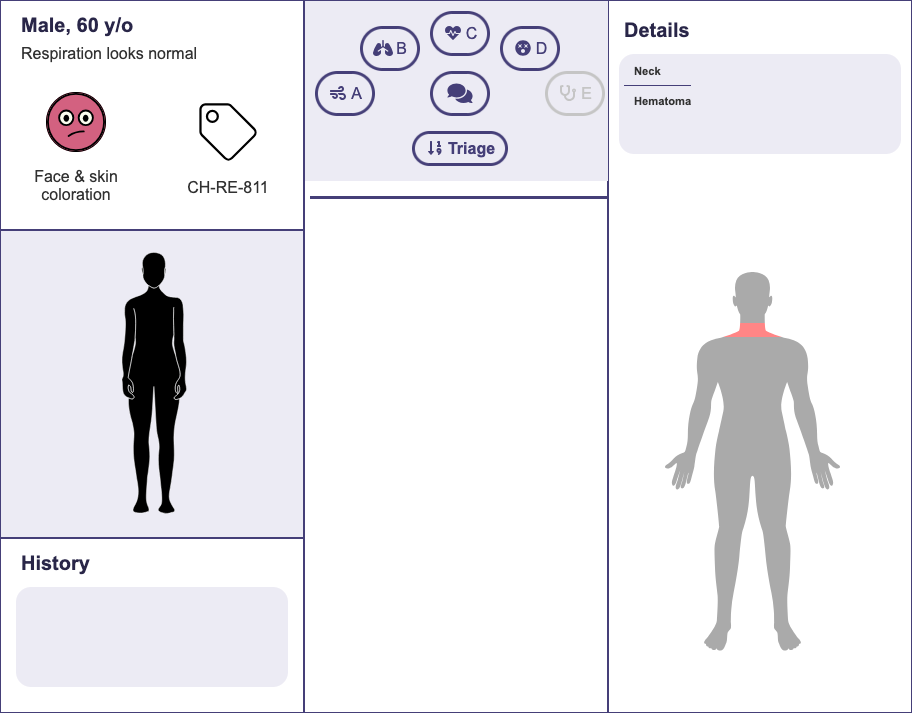

Supplement: Stuby et al. supplementary material [file S1049023X25101568sup001.zip › CH-RE-811.png]

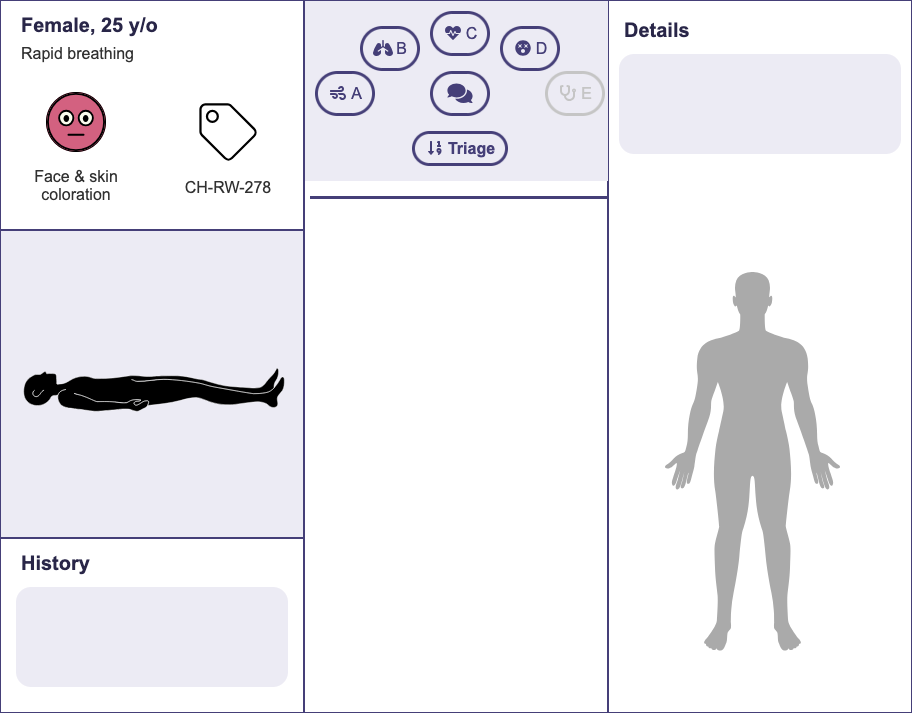

Supplement: Stuby et al. supplementary material [file S1049023X25101568sup001.zip › CH-RW-278.png]

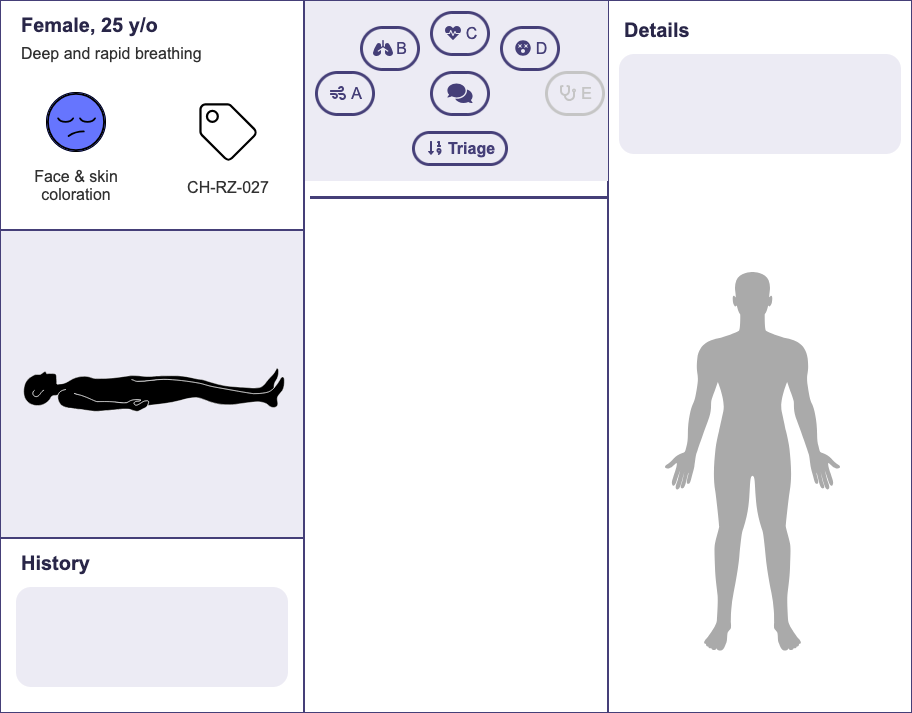

Supplement: Stuby et al. supplementary material [file S1049023X25101568sup001.zip › CH-RZ-027.png]

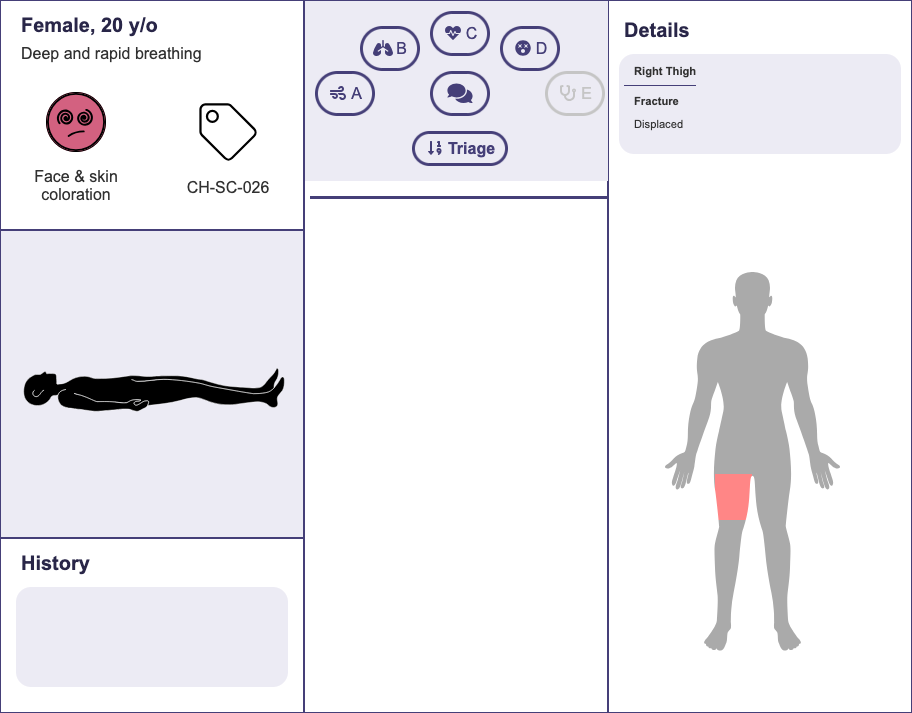

Supplement: Stuby et al. supplementary material [file S1049023X25101568sup001.zip › CH-SC-026.png]

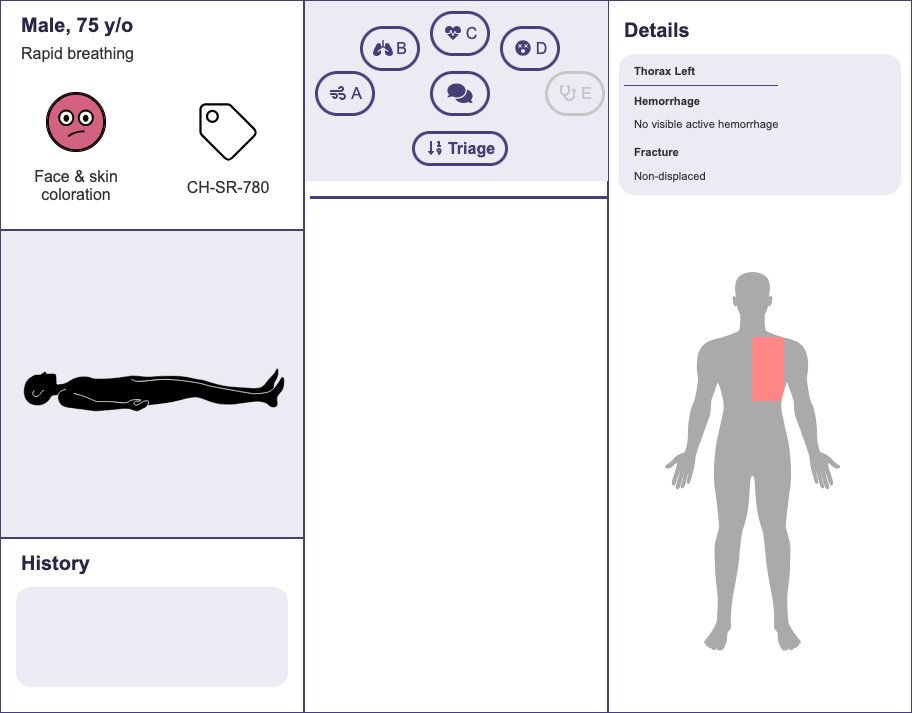

Supplement: Stuby et al. supplementary material [file S1049023X25101568sup001.zip › CH-SR-780.png]

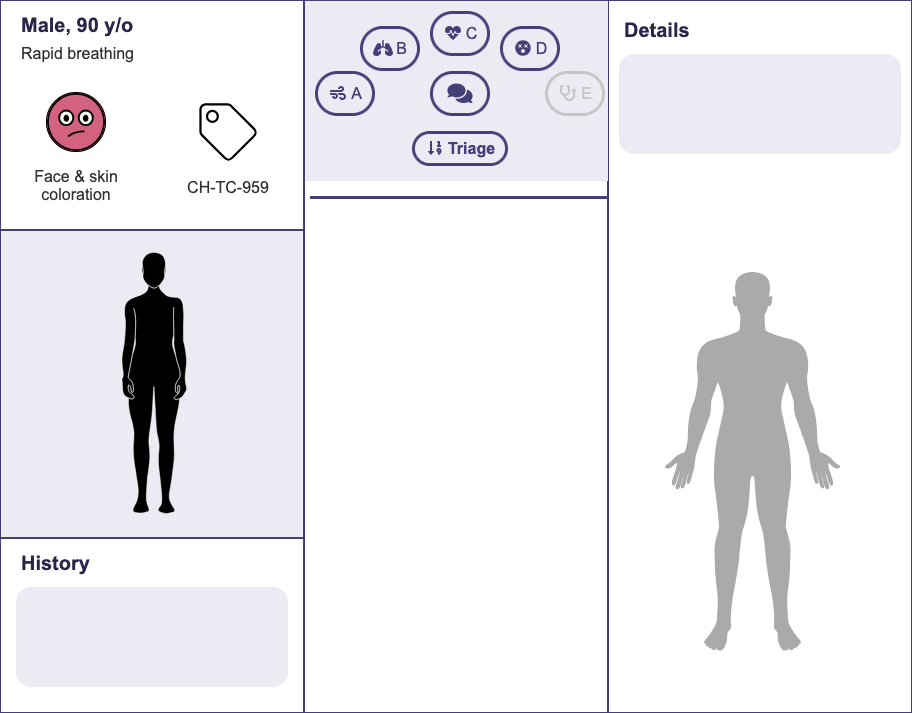

Supplement: Stuby et al. supplementary material [file S1049023X25101568sup001.zip › CH-TC-959.png]

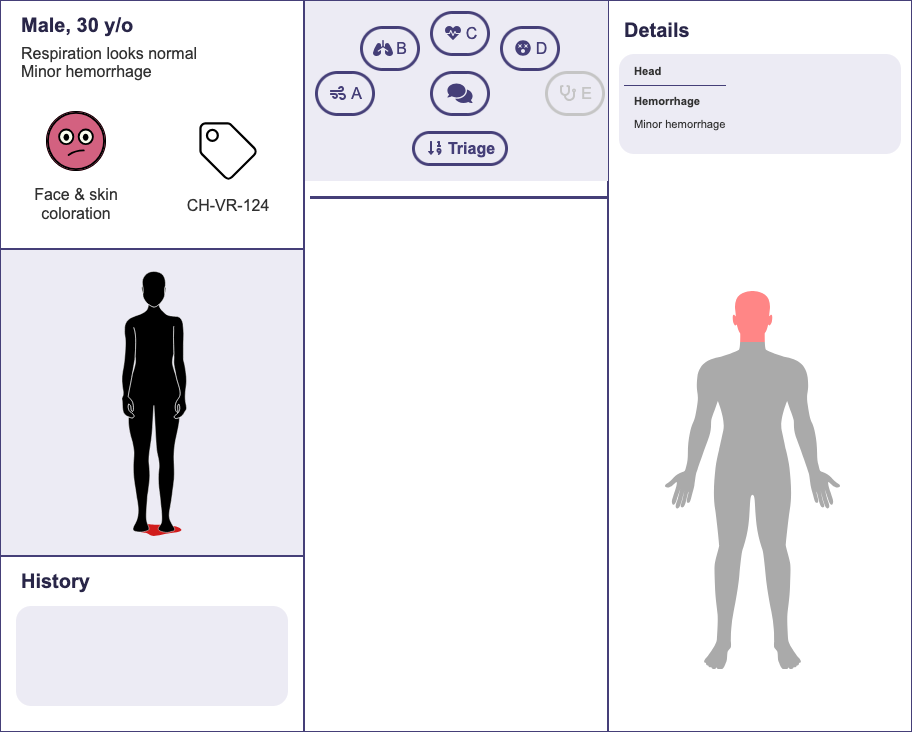

Supplement: Stuby et al. supplementary material [file S1049023X25101568sup001.zip › CH-VR-124.png]

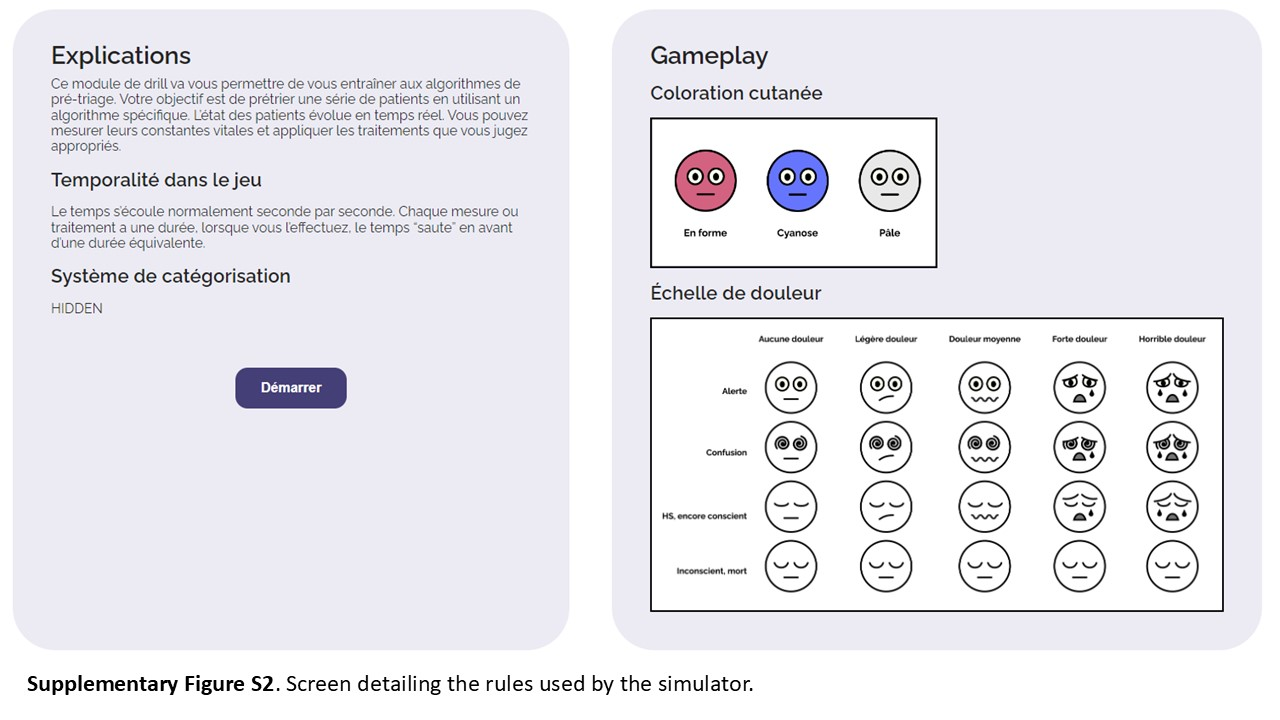

Supplement: Stuby et al. supplementary material [file S1049023X25101568sup001.zip › S2_Figure_S2_-_Rules_screen.tif]
